# Gut Microbiota Mediate Periampullary Cancer Through Extracellular Matrix Proteins: A Causal Relationship Study

**DOI:** 10.1049/syb2.70027

**Published:** 2025-07-21

**Authors:** Zeying Cheng, Liqian Du, Hongxia Zhang, Zhongkun Zhou, Yunhao Ma, Baizhuo Zhang, Lixue Tu, Tong Gong, Zhenzhen Si, Hong Fang, Jianfang Zhao, Peng Chen

**Affiliations:** ^1^ School of Pharmacy Lanzhou University Lanzhou China; ^2^ State Key Laboratory of Applied Organic Chemistry Lanzhou University Lanzhou China; ^3^ Department of Gastroenterology Sanaitang Hospital Lanzhou China; ^4^ Third People's Hospital of Gansu Province Lanzhou China

**Keywords:** bioinformatics, cancer, microorganisms

## Abstract

Recent studies have reported that gut microbiota may play a role in the occurrence and development of digestive system cancers. Periampullary cancer is a relatively rare digestive system cancer which lacks effective targeted therapy and specific drugs. The purpose of this study is to elucidate the relationship between periampullary cancer and gut microbiota. This work collected public genome‐wide association study (GWAS) data from 211 gut microbial taxa and three types of cancer related to periampullary cancer, which were used for two‐sample Mendelian randomisation (MR) analysis. Based on the analysis of differentially expressed genes between periampullary cancer and adjacent normal tissue, extracellular matrix proteins were selected for further multivariable MR analysis. Finally, the Connectivity Map was used to screen potential therapeutic drugs for periampullary cancer. Two‐sample MR results confirmed that nine microbial taxa, *Tyzzerella*, *Alloprevotella*, *Holdemania*, LachnospiraceaeUCG010, *Terrisporobacter*, *Alistipes*, Rikenellaceae, *Anaerofilum* and *Dialister*, were associated with periampullary cancer risk. Multivariable MR discovered extracellular matrix‐related proteins [Collagen alpha‐1(I) chain, Laminin, Fibronectin and Mucin] that may play a role in the association between gut microbiota and periampullary cancer. Finally, the Connectivity Map identified 27 potential candidate drugs. This study can provide theoretical basis for future prevention and diagnostic research on this rare cancer.

## Graphical abstract

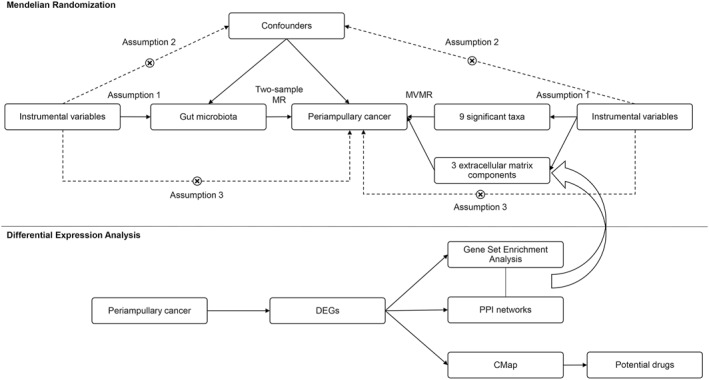

**Abbreviations**
BP: Biological process
CC: Cellular component
CMap: Connectivity Map
DEGs: Differentially expressed genes
ECM: Extracellular matrix
GEO: Gene Expression Omnibus
GO: Gene ontology
GWAS: Genome‐wide association study
ICD‐10: International Classification of Diseases Tenth Revision
IVs: Instrumental variables
IVW: Inverse variance weighted
KEGG: Kyoto Encyclopaedia of Genes and Genomes
LD: Linkage disequilibrium
MF: Molecular function
MR: Mendelian randomisation
MVMR: Multivariable Mendelian randomisation
PDAC: Pancreatic ductal adenocarcinoma
PPI: Protein‐protein interaction
SNPs: Single‐nucleotide polymorphisms
TME: Tumour microenvironment

## 1 Introduction

Periampullary cancer is a malignant tumour that originates within 2 cm of the ampulla of Vater [1], including four different cancers: ampulla of Vater cancer, distal bile duct cancer, pancreatic head cancer, and duodenal cancer [2]. Periampullary cancer is a rare digestive tract tumour accounting for approximately 5% of all gastrointestinal tract malignancies [3]. Most of the periampullary cancers are pancreatic (50%–75%), followed by ampullary (10%–20%), biliary (10%–20%), and duodenal (3%–7%) [4]. Because of different types, the prognosis of periampullary cancer varies and different treatment plans should be adopted. However, due to the low incidence rate of this cancer, the difficulties in obtaining clinical samples lead to the inadequacy of relevant research. Currently, only pancreatoduodenectomy can be used as the standard treatment [5] and there is no consensus on chemotherapy and radiation therapy methods [6]. As for targeted therapy, there are also few research reports and no specific therapeutic drugs available [7, 8, 9, 10].

In recent years, the regulatory role of gut microbiota on tumours has attracted increasing attention. The dysbiosis of gut microbiota can lead to the occurrence of cancer and gut microbiota can regulate tumour activity to interfere with drug efficacy [11]. Based on this, by screening key microbes, we can pave new avenues for the development of cancer diagnosis and treatment methods. Research on the association between colorectal cancer and gut microbiota is the most in‐depth, the gut microbiota is considered a biomarker for colorectal cancer and is used to construct microbial diagnostic models [12]. In addition, faecal microbiota transplantation, probiotic strains, prebiotics, and synbiotics have also been used for the treatment of colorectal cancer [13]. Moreover, gastric cancer is also a well‐known cancer significantly associated with microbiota and *Helicobacter pylori* infection is an important carcinogenic factor [14]. Besides gastrointestinal tumours, gut microbiota can also have an impact on cancer in other parts of the body. Research reports showed that gut microbiota imbalance participated in breast cancer through oestrogen‐dependent and nonoestrogen‐dependent mechanisms [15]. In research on lung cancer, it also found that the microbiota within the tumour may originate from the translocation of gut microbiota [16]. Similarly, the involvement of gut microbiota was found in periampullary cancer. As a type of periampullary cancer, pancreatic ductal adenocarcinoma (PDAC) has a unique tumour microenvironment (TME) in that its dense stroma can destroy tumour vasculature and reduce blood vessel density [17]. The hypoxic microenvironment generated can lead to the block of drug delivery and the reduction of tumour immunogenicity [18]. Recent studies indicated that microbiome originally present in the gut can pass through the Oddi sphincter into the ampulla region and migrate to the pancreas [19], and they may contribute to the tumourigenesis, metastasis and prognosis of PDAC by inducing local inflammation and altering TME [20]. Precisely because there are complex interactions that exist between the microbiome and the TME, including direct contact between cancer cells and microbes as well as indirect action through signalling molecules [21]; further study of the cancer‐associated microbiome could shed new light on cancer treatment.

The extracellular matrix (ECM), a three‐dimensional structural network, is a key component of the TME and plays an important role in regulating cell and tissue function [22, 23]. ECM is composed of various structural proteins, proteoglycans, glycoproteins and glycosaminoglycans. Type I collagen is the most abundant protein in vertebrates and is a major component of the basement membrane, which is assembled into fibres to provide mechanical support for organs and tissues [24]. And collagen *α*1(I) is an essential component of type I collagen trimer. Laminin is one of the main glycoproteins in the basement membrane, involved in cell adhesion and migration, and closely related to tumour angiogenesis and invasion [25]. Fibronectin is an important glycoprotein in the ECM, which regulates cell adhesion, migration, proliferation, and directs the assembly of ECM proteins such as collagen and fibrillin [26, 27].

Mendelian randomisation (MR) is a method to integrate summary data of genome‐wide association study (GWAS) for predicting causality, which uses genetic variations such as single‐nucleotide polymorphism (SNPs) as instrumental variables (IVs) to avoid the impact of confounding factors. To confirm a potential link between gut microbiota and periampullary cancer, we conducted a two‐sample MR analysis. Furthermore, based on the results of Gene set enrichment analysis, Multivariable Mendelian randomisation (MVMR) was used to further explore the role of ECM proteins in this link. Finally, we tried drug repositioning using Connectivity Map (CMap). CMap is a large database established in 2006 to connect drugs, diseases and genes by common gene expression signatures [28]. A query using a set of differentially expressed genes (DEGs) that represent a biological state generates a connectivity score for each drug [29], and if the connectivity score is positive, the small molecule drug promotes the expression of DEGs. In contrast, a negative score indicates that the expression of differential genes is suppressed, and the drug may have a certain therapeutic effect. In the field of adjuvant therapy for periampullary cancer, most studies are retrospective [30]. In the context of the association between cancer and the gut microbiota, traditional research typically employs 16S rRNA sequencing. However, due to the rarity of periampullary cancer, obtaining samples is challenging. Consequently, the limited existing studies have only reported microbiota changes in periampullary cancer patients caused by biliary drainage [31, 32]. Compared with traditional research, the above methods are not restricted by confounding factors and insufficient clinical samples to a considerable extent. Our research results provide evidence for the development of microbiome‐based diagnosis and nonsurgical treatment of periampullary cancer.

## 2 Materials and Methods

### 2.1 Study Design

**FIGURE 1:**
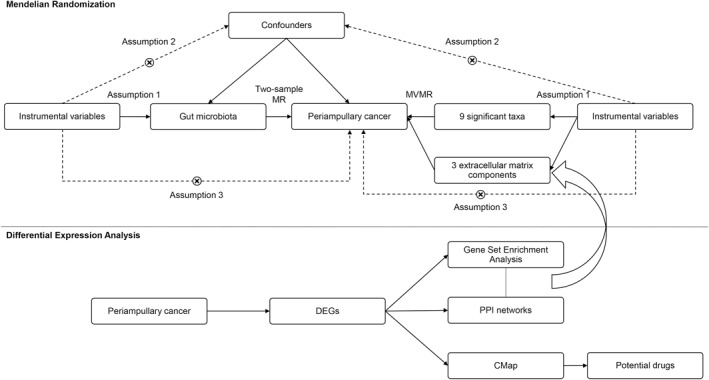
Schematic diagram of Mendelian randomisation and differential expression analysis.

To verify the possible association between gut microbiota and periampullary cancer, two‐sample MR and MVMR analyses were performed. In addition, differential expression analysis was conducted simultaneously, which guided variable selection for MVMR and suggested possible therapeutic drugs. The flow chart of this study is shown in Figure 1.

### 2.2 Data Sources

The data analysed in this paper are from published public databases, details of which are available in the original paper (Table 1). The GWAS data for gut microbiota was derived from large‐scale association analyses by the MiBioGen consortium. The study included 211 microbial taxa (131 genera, 35 families, 20 orders, 16 classes and 9 phyla) and involved 18,340 participants from 24 different cohorts [33]. GWAS summary statistics related to periampullary cancer were sourced from the FinnGen study [34]. Malignant tumours from three different locations (biliary tract, pancreas, and small intestine) were selected as representatives of periampullary cancer. All three sets of data were extracted from OpenGWAS database [35], including 109 cases of biliary tract cancer (ID: finn‐b‐C3_BILIARY_TRACT_EXALLC), 605 cases of pancreatic cancer (finn‐b‐C3_PANCREAS_EXALLC), 252 cases of small intestine cancer (finn‐b‐C3_SMALL_INTESTINE_EXALLC). It is worth noting that the biliary tract cancer we selected is described as malignant neoplasm of other and unspecified parts of biliary tract. In the International Classification of Diseases Tenth Revision (ICD‐10), which was in force at the time, both distal bile duct cancer and ampulla of Vater cancer fell under this classification. Furthermore, we obtained the mRNA expression profiles of periampullary adenocarcinomas and adjacent normal tissues from the Gene Expression Omnibus (GEO) database, focusing on analysing the expression differences of such cancer tissues at the gene level. Tumour samples were collected from 85 patients in this study, of which 49 PDAC, 8 bile duct adenocarcinoma, 8 ampulla of pancreatobiliary type, 7 ampulla of intestinal type, 9 duodenum sample and 12 adjacent normal samples were used for mRNA analysis [36]. Based on the results of the expression profiles analysis, we then explored the potential effects of ECM proteins in microbial carcinogenesis. We retrieved GWAS data for four key ECM components from two human blood plasma proteome studies. Data for collagen alpha‐1(I) chain, laminin and mucin were obtained from a study of 3301 participants with 10,534,735 SNPs [37]. Another study involving 1338 participants with 501,428 SNPs provided data on fibronectin [38]. The results of both studies described above are available from the OpenGWAS database. The populations of the above data are largely independent of each other.

**TABLE 1:** Data sources of this study.

Data type	Trait		Data source	Sample size	Doi	Population	Year
GWAS	Gut microbiota	Phylum	MiBioGen	18,340	https://doi.org/10.1038/s41588‐020‐00763‐1	European (*n* = 13,266), Middle Eastern (*n* = 481), East Asian (*n* = 811), American Hispanic/Latin (*n* = 1097), African American (*n* = 114), multiple ancestries (*n* = 2571)	2021
		Class					
		Order					
		Family					
		Genus					
	Periampullary cancer	Biliary tract cancer	FinnGen	174,115	https://doi.org/10.1101/2022.03.03.22271360	European	2021
		Pancreatic cancer		180,829			2021
		Small intestine cancer		180,959			2021
	ECM protein	Collagen alpha‐1(I) chain	Sun et al.	3301	https://doi.org/10.1038/s41586‐018‐0175‐2	European	2018
		Laminin					
		Mucin					
		Fibronectin	Suhre et al.	1338	https://doi.org/10.1038/ncomms14357	European	2019
Gene expression profiles	Periampullary cancer	PDAC	Sandhu et al.	85	https://doi.org/10.1016/j.molonc.2014.12.002	European	2014
		Biliary tract cancer					
		Pancreatobiliary type ampullary cancer					
		Intestinal type ampullary cancer					
		Duodenum cancer					
		Adjacent normal samples					

### 2.3 Instrumental Variable Selection

The available instrumental variables (IVs) in Mendelian randomisation study must satisfy three assumptions: (1) IVs are strongly correlated with exposure. (2) IVs cannot be associated with any confounding factors. (3) IVs cannot be directly related to the outcome. In order to meet the above three assumptions, we adopted the following steps to screen SNPs. First, SNPs with *p*‐values below the Locus‐wide significance threshold (1 × 10^−5^) were selected to ensure the correlation between IVs and exposure. Second, we obtained independent SNPs (*r* ^2^ < 0.001, clustering distance = 10,000 kb) through linkage disequilibrium (LD) clumping. Then, palindromic SNPs were removed to avoid allelic influence on the results. Finally, we calculated the strength of SNPs using F‐statistic formula, where *F*‐statistic ≥ 10 indicates no weak instrumental bias. The calculation formula is given as follows:

$$F=\frac{\left(N-1-K\right)\times R^{2}}{K\times \left(1-R^{2}\right)}$$

*K* is the number of IVs, *R* ^2^ represents the degree to which the IVs explains the exposure, and *N* is the sample size.

### 2.4 Statistical Analysis

We used six models for two‐sample MR analysis, including Inverse variance weighted (IVW), MR‐Egger, Simple mode, Weighted median, Weighted mode and MR‐PRESSO. IVW was taken as the main evaluation index due to its high accuracy [39], the MR‐Egger intercept test was used to test the potential pleiotropic effect [40], and the MR‐PRESSO corrected the horizontal pleiotropic effect by removing abnormal SNPs [41]. Moreover, Cochran's *Q* statistic was used to assess the heterogeneity of IVs. A Bonferroni correction was performed for multiple testing. *p* < 0.01667 (0.05/3 outcomes) represents statistical significance. The STROBE‐MR checklist is shown in Supporting Information S1: Table S1.

In addition, we performed GEO2R analysis on the mRNA expression profiles of 81 tumour tissue samples and 12 normal tissue samples in the GSE60979 series. The DEGs thresholds were set as adj. *p* value < 0.05 and |logFC| > 2, and 233 up‐regulated genes and 453 down‐regulated genes were collected. Based on DEGs, we performed Gene ontology (GO), Kyoto Encyclopaedia of Genes and Genomes (KEGG) enrichment analysis, and constructed protein‐protein interaction (PPI) network to explore possible tumourigenesis mechanisms. Then, Query CMap database with DEGs for drug repositioning screening and small molecules with connectivity scores below −0.8 were selected as potential drugs.

Finally, we followed the two‐sample MR Parameter setting and conducted MVMR analysis using MVMR‐IVW and MVMR Egger methods to evaluate the vertical pleiotropy effects caused by four ECM components. After obtaining the results of simultaneously incorporating four components, we separately analysed the four proteins with MVMR to reveal their respective effects on cancer.

All MR analyses are implemented based on ‘TwoSampleMR’ (version 0.5.7), ‘MRPRESSO’ (version 1.0), and ‘MendelianRandomization’ (version 0.9.0) packages in R (version 4.3.2). GO and KEGG analyses were performed with the ‘clusterProfiler’ (version 4.10.0) package. STRING database and Cytoscape (version 3.10.1) software are used for constructing and visualising PPI networks.

## 3 Result

### 3.1 The Effect of Gut Microbiota on Periampullary Cancer

We first extracted 14,587 significant SNPs of gut microbiota, and after removing LD, 2965 SNPs were obtained as IVs. The *F*‐statistic of IVs ranges from 16.69 to 95.39, indicating no weak instrumental bias. The palindromic SNPs were subsequently removed, two‐sample MR analysis revealed a statistically significant association of 9 microbial taxa with periampullary cancer (Table 2).

**TABLE 2:** The gut microbiota significantly associated with periampullary cancer evaluated by the IVW method.

Exposure	Outcome	Method	SNP (N)	*p*	OR	OR_lci95	OR_uci95
*Alistipes*	Pancreatic cancer	Inverse variance weighted	13	0.0080	0.3909	0.1952	0.7828
*Terrisporobacter*			5	0.0117	2.3309	1.2070	4.5015
Rikenellaceae			20	0.0142	0.5221	0.3105	0.8778
*Tyzzerella3*	Biliary tract cancer		13	0.0011	3.9394	1.7259	8.9920
*Alloprevotella*			5	0.0075	5.2737	1.5601	17.8270
*Holdemania*			14	0.0085	3.9483	1.4191	10.9853
Lachnospiraceaeucg010			10	0.0094	7.5714	1.6447	34.8542
*Anaerofilum*	Intestine cancer		11	0.0017	0.3950	0.2211	0.7055
*Dialister*			12	0.0143	0.3503	0.1514	0.8109

IVW analysis results showed that increased abundance of Rikenellaceae (OR = 0.52, 95% CI = 0.31–0.88, *p* = 0.0142) and its subordinate genus *Alistipes* (OR = 0.39, 95% CI = 0.20–0.78, *p* = 0.0080) were associated with a reduced risk of pancreatic cancer, whereas *Terrisporobacter* (OR = 2.33, 95% CI = 1.21–4.50, *p* = 0.0117) was associated with an elevated risk. In addition, we found *Tyzzerella3* (OR = 3.94, 95% CI = 1.73–8.99, *p* = 0.0011), *Alloprevotella* (OR = 5.27, 95% CI = 1.56–17.83, *p* = 0.0075), *Holdemania* (OR = 3.95, 95% CI = 1.42–10.99, *p* = 0.0085) and LachnospiraceaeUCG010 (OR = 7.57, 95% CI = 1.64–34.85, *p* = 0.0094) abundance was strongly positive associated with the risk of biliary tract cancer. *Anaerofilum* (OR = 0.40, 95% CI = 0.22–0.71, *p* = 0.0017) and *Dialister* (OR = 0.35, 95% CI = 0.15–0.81, *p* = 0.0143) showed negative associations with the risk of small intestine cancer. Sensitivity analysis showed no evidence of pleiotropy and heterogeneity (all *p*_PRESSO_ > 0.05, all *p*_Egger intercept_ > 0.05, all *p*_Cochran's *Q*_ > 0.05), demonstrating the robustness of MR Results (Table 3). The results of MR Egger, Simple mode, Weighted media, and Weighted mode are provided in Supporting Information S1: Table S2. Scatter plot, forest plot, funnel plot and leave‐one‐out plot are shown in Supporting Information S1: Figure S1–S4.

**TABLE 3:** Sensitivity analysis between gut microbiome and periampullary cancer.

Exposure	Outcome	SNP (N)	*Q*_*P* (IVW)	*Q*_*P* (MR Egger)	*p*_Egger intercept_	*p*_Presso_
*Alistipes*	Pancreatic cancer	13	0.4346	0.3629	0.7380	0.4567
*Terrisporobacter*		5	0.6809	0.5865	0.5876	0.5777
Rikenellaceae		20	0.3822	0.3214	0.9733	0.1523
*Tyzzerella3*	Biliary tract cancer	13	0.7707	0.9288	0.1044	0.7960
*Alloprevotella*		5	0.2023	0.1196	0.8217	0.3930
*Holdemania*		14	0.8084	0.8366	0.2933	0.8663
LachnospiraceaeUCG010		10	0.4097	0.7045	0.0865	0.6357
*Anaerofilum*	Intestine cancer	11	0.8514	0.8819	0.3144	0.9243
*Dialister*		12	0.9216	0.9708	0.2089	0.9510

### 3.2 Differential Gene Analysis in Periampullary Cancer Highlights ECM Proteins

**FIGURE 2:**
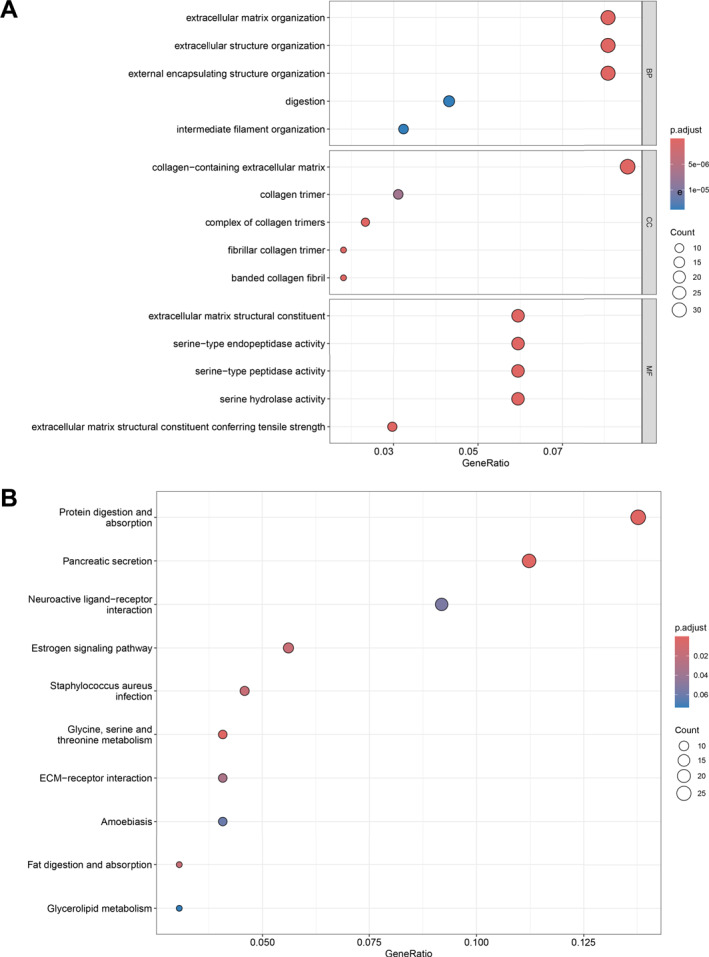
Results of differential gene enrichment analysis. (A) GO enrichment analysis. (B) KEGG pathway analysis.

**FIGURE 3:**
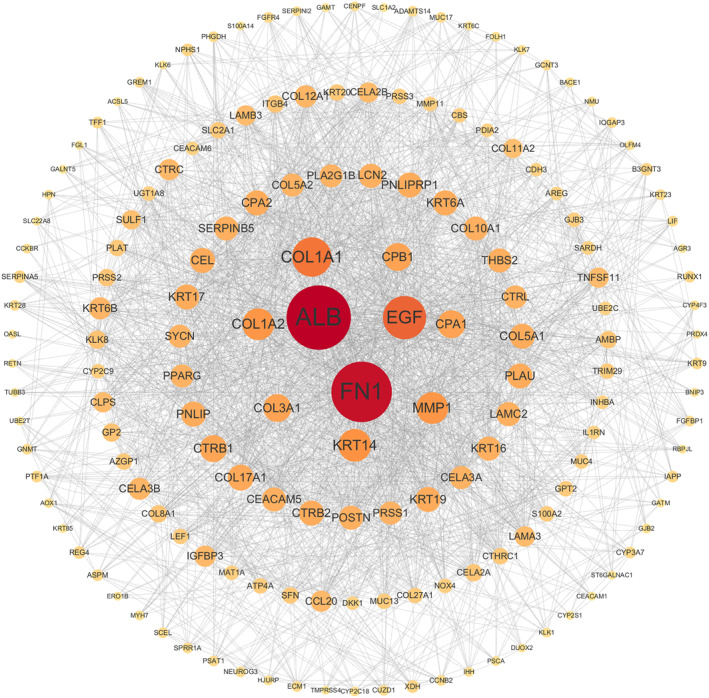
PPI networks constructed based on DEGs. Genes with a degree algorithm score of no less than 10 are displayed. The deeper the colour and the larger the node, the higher the gene score.

A total of 686 DEGs were screened from the mRNA expression profile of periampullary cancer, including 233 up‐regulated genes and 453 down‐regulated genes. GO, KEGG, and PPI networks analyses were used to explore enrichment pathways and search for key proteins, thereby elucidating the effects of these genetic differences on tumourigenesis and progression. The results of GO enrichment analysis (Figure 2A) showed that 10 of the top 15 enrichment pathways in the three modules of cellular component (CC), biological process (BP) and molecular function (MF) were directly related to ECM. Moreover, the serine peptidases indicated by the other three pathways of MF may also be related to the degradation of ECM components [42, 43]. KEGG enrichment analysis (Figure 2B) also revealed the ECM receptor interaction pathway, and we found that the functions of differential genes were mainly concentrated in protein digestion and absorption, pancreatic secretion, neuroactive ligand‐receptor interaction, oestrogen signalling pathway, and *staphylococcus aureus* infection. Then, we use the Degree algorithm in Cytoscape software to score the genes in the constructed PPI network. The top 10 genes are *ALB*, *FN1*, *EGF*, *COL1A1*, *KRT14*, *MMP1*, *COL1A2*, *COL3A1*, *CPA1*, and *CPB1*, 6 of which are closely related to ECM. *FN1*, *COL1A1*, *COL1A2* and *COL3A1* encode ECM structural proteins. *EGF* encodes epidermal growth factor, which can combine with ECM components to regulate cell function [44]. *MMP1*‐encoded matrix metalloproteinase‐1 can degrade ECM proteins [45]. As shown in Figure 3, our results are suggestive of the effect of ECM on periampullary cancer. Table 4 displays the differential expression analysis results of highly differential ECM components (|logFC| > 2.5) between tumour tissues and adjacent normal tissues in periampullary cancer, and it is found that these ECM components are consistently highly expressed in tumour tissues.

**TABLE 4:** Expression differences of core ECM proteins between cancerous and normal tissues.

Gene	*t*	*B*	logFC	Adj. *p* value	*p* value
COL1A1	5.5389	6.5686	3.0544	7.60E‐06	2.78E‐07
COL10A1	4.9216	4.1173	3.0794	5.81E‐05	3.67E‐06
COL17A1	5.8648	7.9211	3.8236	2.58E‐06	6.72E‐08
FN1	5.4015	6.0100	2.5001	1.19E‐05	5.00E‐07
LAMA3	6.3930	10.1828	2.6679	5.22E‐07	6.31E‐09
LAMB3	8.6351	20.3844	3.7225	2.66E‐09	1.50E‐13
LAMC2	7.3891	14.6278	3.0972	3.53E‐08	6.09E‐11
MUC4	4.7165	3.3394	2.5151	1.12E‐04	8.36E‐06
MUC17	4.1428	1.2741	3.8463	6.57E‐04	7.52E‐05

### 3.3 The Role of ECM in the Causal Relationship Between Gut Microbiota and Periampullary Cancer

To verify the potential contribution of ECM in the association between gut microbiota and periampullary cancer, we performed MVMR analysis on four ECM components [collagen alpha‐1(I) chain, laminin, fibronectin and mucin] that showed significant differences in differential expression analysis, with the results presented in Table 5.

**TABLE 5:** MVMR results of four ECM components were adjusted simultaneously.

Exposure	Outcome	Method	Beta	SE	*p*	*Q*_*P*	*p*_Egger intercept_
*Alistipes*	Pancreatic cancer	MVMR_IVW	−0.0067	0.7183	0.9925	0.0920	
		MVMR_Egger	−0.8296	0.9217	0.3681	0.1690	0.1275
*Terrisporobacter*		MVMR_IVW	0.9356	0.7324	0.2014	0.0480	
		MVMR_Egger	1.7831	0.8883	0.0447	0.1241	0.0934
Rikenellaceae		MVMR_IVW	−0.1450	0.6071	0.8113	0.0646	
		MVMR_Egger	−1.1055	0.7590	0.1452	0.0560	0.1492
*Tyzzerella3*	Biliary tract cancer	MVMR_IVW	1.9163	0.9486	0.0434	0.8172	
		MVMR_Egger	1.2743	1.1848	0.2822	0.3658	0.8176
*Alloprevotella*		MVMR_IVW	2.0237	2.9379	0.4909	0.6446	
		MVMR_Egger	2.9540	4.6520	0.5254	0.7965	0.5251
*Holdemania*		MVMR_IVW	−0.6273	1.1313	0.5792	0.5906	
		MVMR_Egger	1.1147	1.4977	0.4567	0.0759	0.7696
LachnospiraceaeUCG010		MVMR_IVW	1.1959	1.1452	0.2963	0.4743	
		MVMR_Egger	1.8836	1.6013	0.2395	0.5345	0.4298
*Anaerofilum*	Intestine cancer	MVMR_IVW	−0.8890	0.4318	0.0395	0.4442	
		MVMR_Egger	−1.1606	0.6108	0.0574	0.5203	0.3996
*Dialister*		MVMR_IVW	−1.5588	0.9425	0.0981	0.7393	
		MVMR_Egger	−0.9347	1.4131	0.5083	0.5534	0.6990

MVMR‐IVW results showed that *p* values of *Alistipes*, Rikenellaceae and *Terrisporobacter* all increased significantly, losing their association with pancreatic cancer. For bile tract cancer, we found increased *p* values of *Alloprevotella*, *Holdemania* and LachnospiraceaeUCG010, but *Tyzzerella3* remained robust. In small intestine cancer, the significance of *Dialister* disappeared, while the significance of *Anaerofilum* was maintained.

In order to understand the respective contributions of the four ECM components, MVMR analyses were performed for each of these components. As shown in Table 6, when using MVMR‐IVW to adjust fibronectin, *Alloprevotella*, *Holdemania*, LachnospiraceaeUCG010, *Tyzzerella3*, Rikenellaceae, *Alistipes*, *Terrisporobacter* and *Dialister* were no longer associated with periampullary cancer. However, when collagen alpha‐1(I) chain was adjusted separately, only the *p* value of *Holdemania* and LachnospiraceaeUCG010 was detected to increase. When laminin was regulated separately, only the *p* value of Rikenellaceae increased, the significant associations of most taxa remained robust. When mucin was included in the analysis, *Terrisporobacter* and Rikenellaceae lost their association with periampullary cancer. We also observed that all taxa for which significant associations disappeared in four components simultaneous adjustment also lost significant associations when only fibronectin was adjusted. No pleiotropy or heterogeneity was found in all MVMR analyses (all *p*_Egger intercept_ > 0.05, all *p*_Cochran's *Q*_ > 0.05).

**TABLE 6:** MVMR results of four ECM components were adjusted separately.

Exposure	ECM components	Outcome	Method	Beta	SE	*p*	*Q*_*P*	*p*_Egger intercept_
*Alistipes*	Collagen alpha‐1(I) chain	Pancreatic cancer	MVMR_IVW	−0.9003	0.3570	0.0117	0.8852	
			MVMR_Egger	−0.7667	0.5170	0.1381	0.8586	0.7209
	Laminin		MVMR_IVW	−0.9447	0.3436	0.0060	0.4755	
			MVMR_Egger	−1.3915	0.6330	0.0279	0.4559	0.4005
	Fibronectin		MVMR_IVW	−0.3219	0.6678	0.6298	0.1526	
			MVMR_Egger	−1.9227	1.2376	0.1203	0.2531	0.1359
	Mucin		MVMR_IVW	−0.8262	0.3997	0.0387	0.1149	
			MVMR_Egger	−1.9039	0.6882	0.0057	0.0605	0.2110
*Terrisporobacter*	Collagen alpha‐1(I) chain		MVMR_IVW	0.7793	0.3173	0.0140	0.9554	
			MVMR_Egger	1.1723	0.4212	0.0054	0.9803	0.1560
	Laminin		MVMR_IVW	0.9350	0.3746	0.0126	0.4869	
			MVMR_Egger	1.5362	0.5054	0.0024	0.6921	0.0763
	Fibronectin		MVMR_IVW	1.6045	0.8336	0.0542	0.2256	
			MVMR_Egger	1.1389	0.9035	0.2075	0.2437	0.2751
	Mucin		MVMR_IVW	0.5438	0.4639	0.2412	0.0583	
			MVMR_Egger	0.7940	0.6361	0.2119	0.5540	0.0478
Rikenellaceae	Collagen alpha‐1(I) chain		MVMR_IVW	−0.5875	0.2778	0.0344	0.7376	
			MVMR_Egger	−0.4958	0.3941	0.2084	0.6979	0.7431
	Laminin		MVMR_IVW	−0.5550	0.2859	0.0523	0.3137	
			MVMR_Egger	−0.7460	0.5033	0.1383	0.2761	0.6421
	Fibronectin		MVMR_IVW	−0.2268	0.7135	0.7505	0.0542	
			MVMR_Egger	−1.9303	1.2087	0.1103	0.1481	0.0971
	Mucin		MVMR_IVW	−0.4841	0.3119	0.1207	0.1078	
			MVMR_Egger	−1.0521	0.5060	0.0376	0.1582	0.1403
*Tyzzerella3*	Collagen alpha‐1(I) chain	Biliary tract cancer	MVMR_IVW	1.3475	0.4161	0.0012	0.5889	
			MVMR_Egger	1.1993	0.5927	0.0430	0.5396	0.7254
	Laminin		MVMR_IVW	1.2896	0.4156	0.0019	0.8661	
			MVMR_Egger	2.1419	0.7336	0.0035	0.9145	0.1586
	Fibronectin		MVMR_IVW	1.2914	0.8918	0.1476	0.7926	
			MVMR_Egger	0.6371	2.0335	0.7540	0.6682	0.7203
	Mucin		MVMR_IVW	1.3673	0.4108	0.0009	0.8795	
			MVMR_Egger	0.9943	0.6802	0.1438	0.4913	0.8646
*Alloprevotella*	Collagen alpha‐1(I) chain		MVMR_IVW	1.3321	0.4984	0.0075	0.2735	
			MVMR_Egger	1.8791	0.8179	0.0216	0.2583	0.3954
	Laminin		MVMR_IVW	1.6391	0.5813	0.0048	0.3542	
			MVMR_Egger	3.5209	1.6165	0.0294	0.4053	0.2150
	Fibronectin		MVMR_IVW	1.7296	1.1228	0.1234	0.8435	
			MVMR_Egger	1.0740	1.6265	0.5091	0.8626	0.5774
	Mucin		MVMR_IVW	1.3609	0.4854	0.0051	0.3912	
			MVMR_Egger	1.1255	1.0251	0.2722	0.7903	0.3018
*Holdemania*	Collagen alpha‐1(I) chain		MVMR_IVW	0.8667	0.5487	0.1142	0.7198	
			MVMR_Egger	1.4667	0.7359	0.0463	0.7507	0.2211
	Laminin		MVMR_IVW	1.2737	0.5351	0.0173	0.8991	
			MVMR_Egger	1.7735	0.8447	0.0358	0.8907	0.4444
	Fibronectin		MVMR_IVW	1.1492	0.9420	0.2225	0.3994	
			MVMR_Egger	0.0039	2.1497	0.9985	0.3264	0.5477
	Mucin		MVMR_IVW	1.4055	0.5545	0.0112	0.7888	
			MVMR_Egger	1.1895	0.8929	0.1828	0.7577	0.7424
LachnospiraceaeUCG010	Collagen alpha‐1(I) chain		MVMR_IVW	1.5554	0.8893	0.0803	0.2992	
			MVMR_Egger	2.0510	1.1828	0.0829	0.2704	0.5173
	Laminin		MVMR_IVW	1.8498	0.7521	0.0139	0.7432	
			MVMR_Egger	1.3539	1.2507	0.2790	0.6997	0.6197
	Fibronectin		MVMR_IVW	1.6704	1.2999	0.1988	0.3369	
			MVMR_Egger	1.3934	2.8993	0.6308	0.2244	0.9121
	Mucin		MVMR_IVW	1.8608	0.7439	0.0124	0.5846	
			MVMR_Egger	1.8684	1.1699	0.1102	0.9933	0.5196
*Anaerofilum*	Collagen alpha‐1(I) chain	Intestine cancer	MVMR_IVW	−0.7016	0.3000	0.0194	0.7176	
			MVMR_Egger	−1.2052	0.4448	0.0067	0.7974	0.1252
	Laminin		MVMR_IVW	−0.9064	0.2904	0.0018	0.6409	
			MVMR_Egger	−0.1590	0.5771	0.7830	0.7355	0.1340
	Fibronectin		MVMR_IVW	−1.2517	0.4152	0.0026	0.5268	
			MVMR_Egger	−1.9047	0.8229	0.0206	0.5086	0.3580
	Mucin		MVMR_IVW	−0.9155	0.3115	0.0033	0.3311	
			MVMR_Egger	−0.1147	0.5775	0.8425	0.1053	0.4307
*Dialister*	Collagen alpha‐1(I) chain		MVMR_IVW	−1.0382	0.4644	0.0254	0.8624	
			MVMR_Egger	−1.9418	0.6885	0.0048	0.9419	0.0754
	Laminin		MVMR_IVW	−1.0913	0.4619	0.0181	0.7462	
			MVMR_Egger	−0.1982	0.8368	0.8128	0.7942	0.2006
	Fibronectin		MVMR_IVW	−0.5254	0.7209	0.4661	0.5206	
			MVMR_Egger	−1.1727	1.2486	0.3476	0.4338	0.5255
	Mucin		MVMR_IVW	−1.1575	0.4344	0.0077	0.6279	
			MVMR_Egger	0.1067	0.7610	0.8885	0.0431	0.8134

### 3.4 Drug Repositioning With CMap

In order to provide a possible solution for the drug therapy of periampullary cancer, we further attempted drug repositioning using CMap. We filtered DEGs with adj. *p* value < 0.05 and |logFC| > 3, obtaining 43 up‐regulated genes and 70 down‐regulated genes for query. As shown in Table 7, 27 drugs with connectivity scores less than −0.8 were obtained and can be used for subsequent research. JNK inhibitor ZG‐10 has the optimal connectivity score. Additionally, it can be observed that HDAC inhibitors have the highest frequency of occurrence among the selected drugs, followed by CDK inhibitors and antibiotics.

**TABLE 7:** Twenty seven candidate drugs from the CMap analysis.

Name	Score	Description	Development stage	Indication
ZG‐10	−0.9697	JNK inhibitor	Preclinical	Not applicable
LY‐303511	−0.9648	Casein kinase inhibitor	Preclinical	Not applicable
TG‐101348	−0.9634	FLT3 inhibitor	Approved	Primary myelofibrosis
PIK‐75	−0.9538	DNA protein kinase inhibitor	Preclinical	Not applicable
Acadesine	−0.9537	AMPK activator	Phase 3	Cardiovascular diseases
ISOX	−0.9492	HDAC inhibitor	Preclinical	Not applicable
CGP‐60474	−0.9460	CDK inhibitor	Preclinical	Not applicable
KIN001‐220	−0.9440	Aurora kinase inhibitor	Preclinical	Not applicable
THM‐I‐94	−0.9341	HDAC inhibitor	Preclinical	Not applicable
Bisindolylmaleimide‐ix	−0.9219	CDK inhibitor	Preclinical	Not applicable
Digitoxigenin	−0.9094	ATPase inhibitor	Preclinical	Not applicable
Alvocidib	−0.8908	CDK inhibitor	Phase 3	Multiple myeloma
Sarmentogenin	−0.8627	ATPase inhibitor	Preclinical	Not applicable
Gatifloxacin	−0.8619	Bacterial DNA gyrase inhibitor	Approved	Bacterial infections
Vorinostat	−0.8615	HDAC inhibitor	Approved	Lymphoma, T‐Cell
Azithromycin	−0.8592	Bacterial 50S ribosomal subunit inhibitor	Approved	Conjunctivitis, bacterial
XMD‐1150	−0.8579	Leucine rich repeat kinase inhibitor	Preclinical	Not applicable
Panobinostat	−0.8562	HDAC inhibitor	Approved	Multiple myeloma
PF‐562271	−0.8513	Focal adhesion kinase inhibitor	Preclinical	Not applicable
Idarubicin	−0.8459	Topoisomerase inhibitor	Approved	Leukaemia, myeloid, acute
Scriptaid	−0.8443	HDAC inhibitor	Preclinical	Not applicable
Verrucarin‐a	−0.8434	Protein synthesis inhibitor	Preclinical	Not applicable
JNK‐9L	−0.8395	JNK inhibitor	Preclinical	Not applicable
BI‐2536	−0.8189	PLK inhibitor	Phase 2	Carcinoma, nonsmall‐cell lung
Cefotaxime	−0.8187	Bacterial cell wall synthesis inhibitor	Approved	Bacterial infections
Alfadolone	−0.8080	GABA receptor agonist	Phase 2	Anaesthetic
Mitoxantrone	−0.8078	Topoisomerase inhibitor	Approved	Prostatic neoplasms

## 4 Discussion

This article is one of the earliest studies to evaluate the causal relationship between gut microbiota and periampullary cancer. It was found that increased abundance of *Tyzzerella3*, *Alloprevotella*, *Holdemania*, LachnospiraceaeUCG010, and *Terrisporobacter* may lead to an increased risk of periampullary cancer, whereas *Alistipes*, Rikenellaceae, *Anaerofilum*, and *Dialister* have a negative association with periampullary cancer. Because of the rarity of periampullary cancer, it is difficult to collect enough samples for traditional observational studies, and MR using genetic variation as instrumental variables can skillfully circumvent this difficulty. Moreover, MR can effectively avoid the interference of confounding factors existing in conventional research. GO, KEGG analysis and PPI networks revealed that DEGs between tumour tissues and adjacent normal tissues were obviously concentrated on ECM‐related pathways whereby we further investigated whether ECM plays a role in the causal relationship between gut microbiota and periampullary cancer. The results suggest that fibronectin may mediate the association between 8 taxa (*Alloprevotella*, *Holdemania*, LachnospiraceaeUCG010, *Tyzzerella3*, Rikenellaceae, *Alistipes*, *Terrisporobacter* and *Dialister*) and periampullary cancer, collagen alpha‐1(I) chain affects *Holdemania* and LachnospiraceaeUCG010, laminin is related to Rikenellaceae and mucin affects *Terrisporobacter* and Rikenellaceae. However, *Anaerofilum* is not affected by these four ECM proteins. In addition, we identified 27 small molecules, providing drug candidates for the establishment of adjuvant therapy for periampullary cancer. Our exploration provides evidence of the association between gut microbiota and periampullary cancer from a genetic perspective, filling the research gap in this field.

There is growing evidence that gut microbiota is involved in the occurrence and development of tumour, but it is usually not the direct cause of cancer. Gut microbiota can regulate the activities of lymphoid organs, and exert effects on the TME through immune mediation. This interaction is defined as the immuno‐oncology‐microbiome axis [46]. However, for microbes within tumours, they regulate tumour progression through three mechanisms: (1) increasing gene mutations, (2) regulating oncogenes or oncogenic pathways, and (3) adjusting the immune system [47]. It is worth noting that intratumour microbes may originate from the translocation of gut microbiota, especially in digestive system cancers. The ampulla of Vater is the junction of bile duct, pancreatic duct and duodenum [48], and there is a greater risk of gut microbiota migration in the tumour around the ampulla. Therefore, the local and remote effects of gut microbiota need to be considered simultaneously in periampullary cancer. Moreover, microbes translocated into tumours can also exert multifaceted influences on the tumour microenvironment, such as modulating cytokine secretion and immune cell function [49]. All the 9 taxa selected are concentrated in Bacillota or Bacteroidota, among which *Terrisporobacter*, *Tyzzerella3*, LachnospiraceaeUCG010 and *Anaerofilum* belong to Eubacteriales, Rikenellaceae, *Alistipes* and *Alloprevotella* belong to Bacteroidales. Therefore, we speculate that Eubacteriales and Bacteroidales may have a higher correlation with periampullary cancer. Mao et al. found that biliary tract cancer patients with high abundance Bacteroidales had better progression free survival [50]. Yang et al. found that collagen alpha‐1(I) chain homotrimer produced by PDAC cancer cells promoted carcinogenic effects through α3β1 integrin, associated with Bacteroidales in the tumour [51], which similarly validated our focus on the ECM. Among the taxa associated with pancreatic cancer that we excavated, *Alistipes*, a genus under the Rikenellaceae family, has been reported to be beneficial bacteria for pancreatitis [52], pancreatic cancer [53] and liver disease [54]. In addition, there is evidence that *Holdemania* is associated with primary biliary cholangitis [55] and pregnancy intrahepatic cholestasis [56], but the direction of the effect is different from our results, and further investigation is needed. To our best knowledge, the correlation between most taxa and periampullary cancer is reported for the first time, which provides a new direction for the study of periampullary cancer.

Our MVMR analysis found that four ECM proteins, including fibronectin, collagen, laminin and mucin, may mediate the effects of gut microbiota on periampullary cancer, suggesting the possibility of a carcinogenic pathway for microbiota‐ECM‐tumour. The effect of ECM on the TME has been widely reported, and it is generally believed that the dysregulation of ECM homoeostasis occurs during the development of cancer, resulting in a series of biochemical reactions that further induce the proliferation and migration of cancer cells [57]. In addition, there is often excessive tissue fibrosis caused by fibrillar collagen accumulation in solid tumours [58], the increase in tumour hardness associated with dense matrix also induces epithelial–mesenchymal transition, which increases the ability of cancer cells to metastasise and induces malignant phenotypes [59, 60]. The high density of ECM also blocks the metastasis of immune cells to the tumour area and compresses the blood vessels to form an oxygen‐deficient environment, resulting in immunosuppressive effects [61, 62]. Increased ECM stiffness disrupts normal tissue architecture by activating and recruiting stromal cells such as CAFs, and exacerbated fibrosis reduces vasculature to compromise drug delivery, collectively driving chemotherapy resistance and limiting immunotherapy applications [63, 64, 65]. It should be emphasised that drug repositioning via CMap based on DEGs cannot comprehensively evaluate the co‐regulatory effects of drugs, immune cells, and ECM. Future single‐cell and spatial transcriptomic studies are strongly warranted. The presence of microbiota in tumours and the effects of gut microbiota on the immune system suggest a possible association between the microbiota and ECM. The microbiota in contact with tumour cells can produce proteases including collagenase, elastase and hyaluronidase, which may have the effect of degrading ECM [66, 67]. These bacterial proteases may induce post‐translational modifications of host ECM proteins [68] and reduce the immune response by disrupting the host cytokine networks [69]. Studies have shown that interactions between bacterial products and ECM components may influence the development and progression of urothelial bladder cancer [70]. Moreover, mucin, an ECM protein, has been used as a biomarker for typing detection of periampullary cancer [1, 71, 72]. The mechanism of the association between gut microbiota, ECM, and periampullary cancer deserves further research in the future.

The typing identification of periampullary cancer is also an essential part of treatment. Ampullary cancer can be divided into intestinal type and pancreaticobiliary type [73], and these two histopathological phenotypes are very similar to duodenal cancer and pancreatic cancer, respectively. Therefore, ampullary cancer is often confused with other periampullary cancer in diagnosis [74, 75, 76]. However, a recent study pointed out that periampullary cancer can be uniformly divided into intestinal type or pancreaticobiliary type in a similar way as ampullary cancer, and cancers of the same type have the same histopathological features and overall survival regardless of the tumour origin site [77]. In the preoperative diagnosis and adjuvant treatment of periampullary cancer, more consideration should be given to the histological type of the tumour rather than the origin site [78]. At present, the main typing methods are HE staining and immunohistochemical staining, which require the acquisition of tissue samples from patients [1]. Studies have shown that 16% of patients undergoing pancreaticoduodenectomy have relevant clinical misdiagnosis [79]. In such a small anatomical site as the ampulla, the site of origin of periampullary cancer usually does not have an impact on the gut microbiota, and its histological type is more likely to lead to differences in the abundance of gut microbiota. We reported the potential effects of gut microbiota abundance on pancreatic cancer, bile tract cancer and small intestine cancer, and the results can provide evidence for developing a new noninvasive faecal microbiota identification method for histopathological types of periampullary cancer, to guide the adjuvant therapy. With the convergence of biomedical and computer technologies, deep learning may offer potential solutions to this challenge, which will also be our future research trend [80, 81, 82, 83].

Our study has some limitations: (1) Most of our GWAS data are derived from European populations, so extrapolating our results to other populations may not be universally applicable. (2) The GWAS data for the three cancer groups we selected were derived from normal pancreatic, bile tract and small intestine cancer, rather than the specific source of periampullary tissue within 2 cm of the ampulla, which may lead to certain bias. (3) Our MVMR analysis focused on plasma ECM proteins, whereas correlations at the tissue level call for further validation.

## 5 Conclusions

In summary, we conducted MR studies and differential expression analysis using publicly available databases. This work demonstrated a causal relationship between gut microbiota and periampullary cancer, revealed that potential role for ECM proteins in this crosstalk, and provided the potential drug candidates for periampullary cancer. These findings warrant further research to explore the potential clinical application of microbiome related technologies. It could help expand our understanding of periampullary cancer and guide the development of new drugs and diagnostic methods.

## Author Contributions

**Zeying Cheng:** conceptualization, data curation, formal analysis, writing – original draft, writing – review and editing. **Liqian Du:** data curation, formal analysis, writing – review and editing. **Hongxia Zhang:** conceptualization, methodology, project administration. **Zhongkun Zhou:** investigation, methodology, writing – review and editing. **Yunhao Ma:** investigation, writing – review and editing. **Baizhuo Zhang:** investigation, writing – review and editing. **Lixue Tu:** data curation. **Tong Gong:** data curation. **Zhenzhen Si:** data curation. **Hong Fang:** data curation. **Jianfang Zhao:** conceptualization, methodology, project administration. **Peng Chen:** conceptualization, funding acquisition, project administration, supervision, writing – review and editing. All authors discussed the results and approved the manuscript.

## Ethics Statement

This study was conducted based on publicly available databases that have been approved by ethical review committees. Therefore, no new approval by the ethical review committee is required.

## Consent

The authors have nothing to report.

## Conflicts of Interest

The authors declare no conflicts of interest.

## Supporting information

Supporting Information S1

## Data Availability

The data used in this study can be obtained from MiBioGen database (https://mibiogen.gcc.rug.nl/), OpenGWAS database (https://gwas.mrcieu.ac.uk/), and GEO database (https://www.ncbi.nlm.nih.gov/geo/query/acc.cgi?acc=GSE60979).
