# Supplementary material for: Gut Microbiota Mediate Periampullary Cancer Through Extracellular Matrix Proteins: A Causal Relationship Study
Source: IET Syst Biol. 2025 Jul 21;19(1):e70027. doi: 10.1049/syb2.70027 (PMC12279554; doi:10.1049/syb2.70027)

**Supplementary files**

Table S1. Results of the STROBE-MR checklist in Mendelian randomization study.

| **Item No.** | **Section** | **Checklist item** | **Page No.** | **Relevant text from manuscript** |
| --- | --- | --- | --- | --- |
| 1 | **TITLE and ABSTRACT** | Indicate Mendelian randomization (MR) as the study’s design in the title and/or the abstract if that is a main purpose of the study | 1 | Gut microbiota mediates periampullary cancer through extracellular matrix proteins: A rare cancer study based on Mendelian randomization and differential expression analysis |
|  | **INTRODUCTION** |  |  |  |
| 2 | **Background** | Explain the scientific background and rationale for the reported study. What is the exposure? Is a potential causal relationship between exposure and outcome plausible? Justify why MR is a helpful method to address the study question | 5-7 | In recent years, the regulatory role of gut microbiota on tumors has attracted increasing attention.  The dysbiosis of gut microbiota can lead to the occurrence of cancer, and gut microbiota can regulate tumor activity to interfere with drug efficacy.  Precisely because there are complex interactions that exist between the microbiome and the TME, including direct contact between cancer cells and microbes as well as indirect action through signaling molecules, further study of the cancer-associated microbiome could shed new light on cancer treatment.  Mendelian randomization (MR) is a method to integrate summary data of genome-wide association study (GWAS) for predicting causality, which uses genetic variations such as single-nucleotide polymorphism (SNPs) as instrumental variables (IVs) to avoid the impact of confounding factors. |
| 3 | **Objectives** | State specific objectives clearly, including pre-specified causal hypotheses (if any). State that MR is a method that, under specific assumptions, intends to estimate causal effects | 7 | To confirm a potential link between gut microbiota and periampullary cancer, we conducted a two-samples MR analysis. Furthermore, based on the results of Gene set enrichment analysis, Multivariable Mendelian randomization (MVMR) was used to further explore the role of ECM proteins in this link.  Mendelian randomization (MR) is a method to integrate summary data of genome-wide association study (GWAS) for predicting causality, which uses genetic variations such as single-nucleotide polymorphism (SNPs) as instrumental variables (IVs) to avoid the impact of confounding factors. |
|  | **METHODS** |  |  |  |
| 4 | **Study design and data sources** | Present key elements of the study design early in the article. Consider including a table listing sources of data for all phases of the study. For each data source contributing to the analysis, describe the following: |  |  |
|  | a) | Setting: Describe the study design and the underlying population, if possible. Describe the setting, locations, and relevant dates, including periods of recruitment, exposure, follow-up, and data collection, when available. | 8 | The data analyzed in this paper are from published public databases, details of which are available in the original paper (Table 1). |
|  | b) | Participants: Give the eligibility criteria, and the sources and methods of selection of participants. Report the sample size, and whether any power or sample size calculations were carried out prior to the main analysis | 8 | The data analyzed in this paper are from published public databases, details of which are available in the original paper (Table 1). |
|  | c) | Describe measurement, quality control and selection of genetic variants | 8 | The data analyzed in this paper are from published public databases, details of which are available in the original paper (Table 1). |
|  | d) | For each exposure, outcome, and other relevant variables, describe methods of assessment and diagnostic criteria for diseases | 8 | The data analyzed in this paper are from published public databases, details of which are available in the original paper (Table 1). |
|  | e) | Provide details of ethics committee approval and participant informed consent, if relevant | 8 | The data analyzed in this paper are from published public databases, details of which are available in the original paper (Table 1). |
| 5 | **Assumptions** | Explicitly state the three core IV assumptions for the main analysis (relevance, independence and exclusion restriction) as well assumptions for any additional or sensitivity analysis | 9 | The available instrumental variables (IVs) in Mendelian randomization study must satisfy three assumptions: (1) IVs are strongly correlated with exposure; (2) IVs cannot be associated with any confounding factors; (3) IVs cannot be directly related to the outcome. |
| 6 | **Statistical methods: main analysis** | Describe statistical methods and statistics used |  |  |
|  | a) | Describe how quantitative variables were handled in the analyses (i.e., scale, units, model) | 8 | The data analyzed in this paper are from published public databases, details of which are available in the original paper (Table 1). |
|  | b) | Describe how genetic variants were handled in the analyses and, if applicable, how their weights were selected | 9 | First, SNPs with P-values below the Locus-wide significance threshold (1 × 10^-5^) were selected to ensure the correlation between IVs and exposure. Second, we obtained independent SNPs (r2 < 0.001, clustering distance = 10000kb) through linkage disequilibrium (LD) clumping. Then, palindromic SNPs were removed to avoid allelic influence on the results. Finally, we calculated the strength of SNPs using F-statistic formula, where F-statistic ≥ 10 indicates no weak instrumental bias. |
|  | c) | Describe the MR estimator (e.g. two-stage least squares, Wald ratio) and related statistics. Detail the included covariates and, in case of two-sample MR, whether the same covariate set was used for adjustment in the two samples | 10 | We used 6 models for two-sample MR analysis, including Inverse variance weighted (IVW), MR-Egger, Simple mode, Weighted median, Weighted mode and MR-PRESSO. |
|  | d) | Explain how missing data were addressed | 8 | The data analyzed in this paper are from published public databases, details of which are available in the original paper (Table 1). |
|  | e) | If applicable, indicate how multiple testing was addressed | 10 | A Bonferroni correction was performed for multiple testing. p < 0.01667 (0.05/3 outcomes) represents statistical significance. |
| 7 | **Assessment of assumptions** | Describe any methods or prior knowledge used to assess the assumptions or justify their validity | 9 | Finally, we calculated the strength of SNPs using F-statistic formula, where F-statistic ≥ 10 indicates no weak instrumental bias. |
| 8 | **Sensitivity analyses and additional analyses** | Describe any sensitivity analyses or additional analyses performed (e.g. comparison of effect estimates from different approaches, independent replication, bias analytic techniques, validation of instruments, simulations) | 10 | IVW was taken as the main evaluation index due to its high accuracy, the MR-Egger intercept test was used to test the potential pleiotropic effect, and the MR-PRESSO corrected the horizontal pleiotropic effect by removing abnormal SNPs. Moreover, Cochran's Q statistic was used to assess the heterogeneity of IVs. |
| 9 | **Software and pre-registration** |  |  |  |
|  | a) | Name statistical software and package(s), including version and settings used | 11 | All MR analyses are implemented based on “TwoSampleMR” (version 0.5.7), “MRPRESSO” (version 1.0), and “MendelianRandomization” (version 0.9.0) packages in R (version 4.3.2). GO and KEGG analyses were performed with the “clusterProfiler” (version 4.10.0) package. STRING database and Cytoscape (version 3.10.1) software are used for constructing and visualizing PPI networks. |
|  | b) | State whether the study protocol and details were pre-registered (as well as when and where) | 19 | This study was conducted based on publicly available databases that have been approved by ethical review committees. Therefore, no new approval by the ethical review committee is required. |
|  | **RESULTS** |  |  |  |
| 10 | **Descriptive data** |  |  |  |
|  | a) | Report the numbers of individuals at each stage of included studies and reasons for exclusion. Consider use of a flow diagram | 8 | The data analyzed in this paper are from published public databases, details of which are available in the original paper (Table 1). |
|  | b) | Report summary statistics for phenotypic exposure(s), outcome(s), and other relevant variables (e.g. means, SDs, proportions) | 8-9 | The GWAS data for gut microbiota was derived from large-scale association analyses by the MiBioGen consortium. The study included 211 microbial taxa (131 genera, 35 families, 20 orders, 16 classes and 9 phyla) and involved 18,340 participants from 24 different cohorts......Data for collagen alpha-1(I) chain and laminin were obtained from a study of 3,301 participants with 10,534,735 SNPs. Another study involving 1338 participants with 501,428 SNPs provided data on fibronectin. The results of both studies described above are available from the OpenGWAS database. |
|  | c) | If the data sources include meta-analyses of previous studies, provide the assessments of heterogeneity across these studies | 8 | The data analyzed in this paper are from published public databases, details of which are available in the original paper (Table 1). |
|  | d) | For two-sample MR:  i.  Provide justification of the similarity of the genetic variant-exposure associations between the exposure and outcome samples  ii.  Provide information on the number of individuals who overlap between the exposure and outcome studies | 9 | First, SNPs with P-values below the Locus-wide significance threshold (1 × 10-5) were selected to ensure the correlation between IVs and exposure. Second, we obtained independent SNPs (r2 < 0.001, clustering distance = 10000kb) through linkage disequilibrium (LD) clumping. Then, palindromic SNPs were removed to avoid allelic influence on the results. Finally, we calculated the strength of SNPs using F-statistic formula, where F-statistic ≥ 10 indicates no weak instrumental bias.  The populations of the above data are largely independent of each other. |
| 11 | **Main results** |  |  |  |
|  | a) | Report the associations between genetic variant and exposure, and between genetic variant and outcome, preferably on an interpretable scale | 11 | We first extracted 14,587 significant SNPs of gut microbiota, and after removing LD, 2965 SNPs were obtained as IVs. The F-statistic of IVs ranges from 16.69 to 95.39, indicating no weak instrumental bias. |
|  | b) | Report MR estimates of the relationship between exposure and outcome, and the measures of uncertainty from the MR analysis, on an interpretable scale, such as odds ratio or relative risk per SD difference | 11 | IVW analysis results showed that increased abundance of Rikenellaceae (OR = 0.52, 95% CI = 0.31 - 0.88, P = 0.0142) and its subordinate genus Alistipes (OR = 0.39, 95% CI = 0.20 - 0.78, P = 0.0080) were associated with a reduced risk of pancreatic cancer, whereas Terrisporobacter (OR = 2.33, 95% CI = 1.21 - 4.50, P = 0.0117) was associated with an elevated risk. |
|  | c) | If relevant, consider translating estimates of relative risk into absolute risk for a meaningful time period | No Applicable |  |
|  | d) | Consider plots to visualize results (e.g. forest plot, scatterplot of associations between genetic variants and outcome versus between genetic variants and exposure) | 12 | Scatter plot, forest plot, funnel plot and leave-one-out plot are shown in Figure S1-S4. |
| 12 | **Assessment of assumptions** |  |  |  |
|  | a) | Report the assessment of the validity of the assumptions | 12 | Sensitivity analysis showed no evidence of pleiotropy and heterogeneity (all P PRESSO > 0.05, all P Egger intercept > 0.05, all P Cochran’s Q > 0.05), demonstrating the robustness of MR Results (Table 3). |
|  | b) | Report any additional statistics (e.g., assessments of heterogeneity across genetic variants, such as *I^2^*, Q statistic or E-value) | 13 | No pleiotropy or heterogeneity was found in all MVMR analyses (all P Egger intercept > 0.05, all P Cochran’s Q > 0.05). |
| 13 | **Sensitivity analyses and additional analyses** |  |  |  |
|  | a) | Report any sensitivity analyses to assess the robustness of the main results to violations of the assumptions | 11-12 | Sensitivity analysis showed no evidence of pleiotropy and heterogeneity (all P PRESSO > 0.05, all P Egger intercept > 0.05, all P Cochran’s Q > 0.05), demonstrating the robustness of MR Results (Table 3). |
|  | b) | Report results from other sensitivity analyses or additional analyses | 14 | No pleiotropy or heterogeneity was found in all MVMR analyses (all P Egger intercept > 0.05, all P Cochran’s Q > 0.05). |
|  | c) | Report any assessment of direction of causal relationship (e.g., bidirectional MR) | 13 | As shown in Table 6, when using MVMR-IVW to adjust fibronectin, Alloprevotella, Holdemania, LachnospiraceaeUCG010, Tyzzerella3, Rikenellaceae, Alistipes and Dialister were no longer associated with periampullary cancer. |
|  | d) | When relevant, report and compare with estimates from non-MR analyses | 16 | Therefore, we speculate that Eubacteriales and Bacteroidales may have a higher correlation with periampullary carcinoma. Mao et al. found that biliary tract cancer patients with high abundance Bacteroidales had better progression free survival. Yang et al. found that collagen alpha-1(I) chain homotrimer produced by PDAC cancer cells promoted carcinogenic effects through α3β1 integrin, associated with Bacteroidales in the tumor, which similarly validated our focus on the ECM. |
|  | e) | Consider additional plots to visualize results (e.g., leave-one-out analyses) | 12 | Scatter plot, forest plot, funnel plot and leave-one-out plot are shown in Figure S1-S4. |
|  | **DISCUSSION** |  |  |  |
| 14 | **Key results** | Summarize key results with reference to study objectives | 18 | This work demonstrated a causal relationship between gut microbiota and periampullary cancer, revealed that potential role for ECM proteins in this crosstalk, and provided the potential drug candidates for periampullary cancer. |
| 15 | **Limitations** | Discuss limitations of the study, taking into account the validity of the IV assumptions, other sources of potential bias, and imprecision. Discuss both direction and magnitude of any potential bias and any efforts to address them | 19 | Our study has some limitations: (1) Most of our GWAS data are derived from European populations, so extrapolating our results to other populations may not be universally applicable. (2) The GWAS data for the three cancer groups we selected were derived from normal pancreatic, bile tract and small intestine cancer, rather than the specific source of periampullary tissue within 2 cm of the ampulla, which may lead to certain bias. (3) Our MVMR analysis focused on plasma ECM proteins, ‌while‌ correlations at the tissue level ‌call for further validation‌. |
| 16 | **Interpretation** |  |  |  |
|  | a) | Meaning: Give a cautious overall interpretation of results in the context of their limitations and in comparison with other studies | 18 | We reported the potential effects of gut microbiota abundance on pancreatic cancer, bile tract cancer and small intestine cancer, and the results can provide evidence for developing a new non-invasive fecal microbiota identification method for histopathological types of periampullary cancer, to guide the adjuvant therapy. |
|  | b) | Mechanism: Discuss underlying biological mechanisms that could drive a potential causal relationship between the investigated exposure and the outcome, and whether the gene-environment equivalence assumption is reasonable. Use causal language carefully, clarifying that IV estimates may provide causal effects only under certain assumptions | 16 | Our MVMR analysis found that three ECM proteins, including fibronectin, collagen, and laminin, may mediate the effects of gut microbiota on periampullary carcinoma, suggesting the possibility of a carcinogenic pathway for microbiota-ECM-tumor. |
|  | c) | Clinical relevance: Discuss whether the results have clinical or public policy relevance, and to what extent they inform effect sizes of possible interventions | 18 | We reported the potential effects of gut microbiota abundance on pancreatic cancer, bile tract cancer and small intestine cancer, and the results can provide evidence for developing a new non-invasive fecal microbiota identification method for histopathological types of periampullary cancer, to guide the adjuvant therapy. |
| 17 | **Generalizability** | Discuss the generalizability of the study results (a) to other populations, (b) across other exposure periods/timings, and (c) across other levels of exposure | 19 | Our study has some limitations: (1) Most of our GWAS data are derived from European populations, so extrapolating our results to other populations may not be universally applicable. (2) The GWAS data for the three cancer groups we selected were derived from normal pancreatic, bile tract and small intestine cancer, rather than the specific source of periampullary tissue within 2 cm of the ampulla, which may lead to certain bias. (3) Our MVMR analysis focused on plasma ECM proteins, ‌while‌ correlations at the tissue level ‌call for further validation‌. |
|  | **OTHER INFORMATION** |  |  |  |
| 18 | **Funding** | Describe sources of funding and the role of funders in the present study and, if applicable, sources of funding for the databases and original study or studies on which the present study is based | 2 | This work was supported by Gansu Provincial Science and Technology Major Project (Grant No. 24ZDFA001), The Lanzhou Municipal Science and Technology Program (Grant Nos. 2024-8-27, 2024-8-30, 2024-4-2) and the College Students' Innovation and Entrepreneurship Program of Lanzhou University, China (Grant Nos. 20250260006, 20250260016 and 20250260020). |
| 19 | **Data and data sharing** | Provide the data used to perform all analyses or report where and how the data can be accessed, and reference these sources in the article. Provide the statistical code needed to reproduce the results in the article, or report whether the code is publicly accessible and if so, where | 2 | The data used in this study can be obtained from MiBioGen database (https://mibiogen.gcc.rug.nl/), OpenGWAS database (https://gwas.mrcieu.ac.uk/), and GEO database (https://www.ncbi.nlm.nih.gov/geo/query/acc.cgi?acc=GSE60979). |
| 20 | **Conflicts of Interest** | All authors should declare all potential conflicts of interest | 2 | The authors declare that they have no competing interests. |

This checklist is copyrighted by the Equator Network under the Creative Commons Attribution 3.0 Unported (CC BY 3.0) license.

1. Skrivankova VW, Richmond RC, Woolf BAR, Yarmolinsky J, Davies NM, Swanson SA, et al. Strengthening the Reporting of Observational Studies in Epidemiology using Mendelian Randomization (STROBE-MR) Statement. JAMA. 2021;under review.

2. Skrivankova VW, Richmond RC, Woolf BAR, Davies NM, Swanson SA, VanderWeele TJ, et al. Strengthening the Reporting of Observational Studies in Epidemiology using Mendelian Randomisation (STROBE-MR): Explanation and Elaboration. BMJ. 2021;375:n2233.

Table S2. The gut microbiota significantly associated with periampullary cancer evaluated by IVW, MR-Egger, Simple mode, Weighted median and Weighted mode.

| **Exposure** | **Outcome** | **Method** | **SNP (N)** | **BETA** | **SE** | **P** |
| --- | --- | --- | --- | --- | --- | --- |
| *Alistipes* | Pancreatic cancer | Inverse variance weighted | 13 | -0.9393 | 0.3543 | 0.0080 |
|  |  | Weighted median | 13 | -1.2314 | 0.4823 | 0.0107 |
|  |  | Weighted mode | 13 | -1.4149 | 0.9040 | 0.1435 |
|  |  | Simple mode | 13 | -1.3840 | 0.8846 | 0.1437 |
|  |  | MR Egger | 13 | -0.3535 | 1.7466 | 0.8433 |
| *Terrisporobacter* |  | Inverse variance weighted | 5 | 0.8463 | 0.3358 | 0.0117 |
|  |  | Weighted median | 5 | 0.9729 | 0.4381 | 0.0264 |
|  |  | Weighted mode | 5 | 1.0052 | 0.5684 | 0.1517 |
|  |  | Simple mode | 5 | 0.9843 | 0.5665 | 0.1573 |
|  |  | MR Egger | 5 | 1.4302 | 1.0210 | 0.2558 |
| Rikenellaceae |  | Inverse variance weighted | 20 | -0.6499 | 0.2651 | 0.0142 |
|  |  | Weighted median | 20 | -0.6947 | 0.3659 | 0.0576 |
|  |  | Simple mode | 20 | -1.0568 | 0.6522 | 0.1216 |
|  |  | Weighted mode | 20 | -0.7713 | 0.5640 | 0.1874 |
|  |  | MR Egger | 20 | -0.6223 | 0.8582 | 0.4777 |
| *Tyzzerella3* | Biliary tract cancer | Inverse variance weighted | 13 | 1.3710 | 0.4211 | 0.0011 |
|  |  | Weighted median | 13 | 1.5630 | 0.5903 | 0.0081 |
|  |  | MR Egger | 13 | 5.5767 | 2.4131 | 0.0412 |
|  |  | Simple mode | 13 | 2.2280 | 0.9995 | 0.0457 |
|  |  | Weighted mode | 13 | 2.0418 | 0.9368 | 0.0499 |
| *Alloprevotella* |  | Inverse variance weighted | 5 | 1.6627 | 0.6214 | 0.0075 |
|  |  | Weighted median | 5 | 1.5720 | 0.7316 | 0.0317 |
|  |  | Weighted mode | 5 | 1.6349 | 0.9432 | 0.1581 |
|  |  | Simple mode | 5 | 1.6228 | 0.9429 | 0.1604 |
|  |  | MR Egger | 5 | 0.1591 | 6.1592 | 0.9810 |
| *Holdemania* |  | Inverse variance weighted | 14 | 1.3733 | 0.5221 | 0.0085 |
|  |  | Weighted median | 14 | 1.5878 | 0.6873 | 0.0209 |
|  |  | Simple mode | 14 | 1.7917 | 1.1011 | 0.1277 |
|  |  | Weighted mode | 14 | 1.6892 | 1.1125 | 0.1529 |
|  |  | MR Egger | 14 | -0.2109 | 1.5329 | 0.8929 |
| LachnospiraceaeUCG010 |  | Inverse variance weighted | 10 | 2.0244 | 0.7790 | 0.0094 |
|  |  | Weighted median | 10 | 1.9810 | 1.0893 | 0.0690 |
|  |  | Weighted mode | 10 | 2.6223 | 1.7781 | 0.1744 |
|  |  | Simple mode | 10 | 2.6223 | 1.8806 | 0.1967 |
|  |  | MR Egger | 10 | -2.2932 | 2.3389 | 0.3556 |
| *Anaerofilum* | Intestine cancer | Inverse variance weighted | 11 | -0.9289 | 0.2959 | 0.0017 |
|  |  | Weighted median | 11 | -0.9598 | 0.4013 | 0.0168 |
|  |  | Weighted mode | 11 | -0.9514 | 0.5566 | 0.1182 |
|  |  | Simple mode | 11 | -0.9828 | 0.5963 | 0.1304 |
|  |  | MR Egger | 11 | 0.7480 | 1.6013 | 0.6515 |
| *Dialister* |  | Inverse variance weighted | 12 | -1.0488 | 0.4282 | 0.0143 |
|  |  | Weighted median | 12 | -1.0431 | 0.5360 | 0.0517 |
|  |  | Weighted mode | 12 | -1.7609 | 0.9392 | 0.0876 |
|  |  | Simple mode | 12 | -1.8242 | 1.0262 | 0.1031 |
|  |  | MR Egger | 12 | 1.3174 | 1.8130 | 0.4841 |

**Figure Captions**

Figure S1. Scatter plot of association between Rikenellaceae and pancreatic cancer. The slope represents the causal effect evaluated by the corresponding model, and the intercept can show pleiotropy.

Figure S2. Forest plot of the causal association between Rikenellaceae and pancreatic cancer. Each black horizontal line represents the result based on a single SNP, and the red line is the result considering all SNPs.

Figure S3. Leave-one-out plot of the causal association between Rikenellaceae and pancreatic cancer. SNPs are eliminated one by one to determine whether a SNP makes a significant difference to the result.

Figure S4. Funnel plot of the causal association between Rikenellaceae and pancreatic cancer for assessing the degree of bias.

Figure S1.


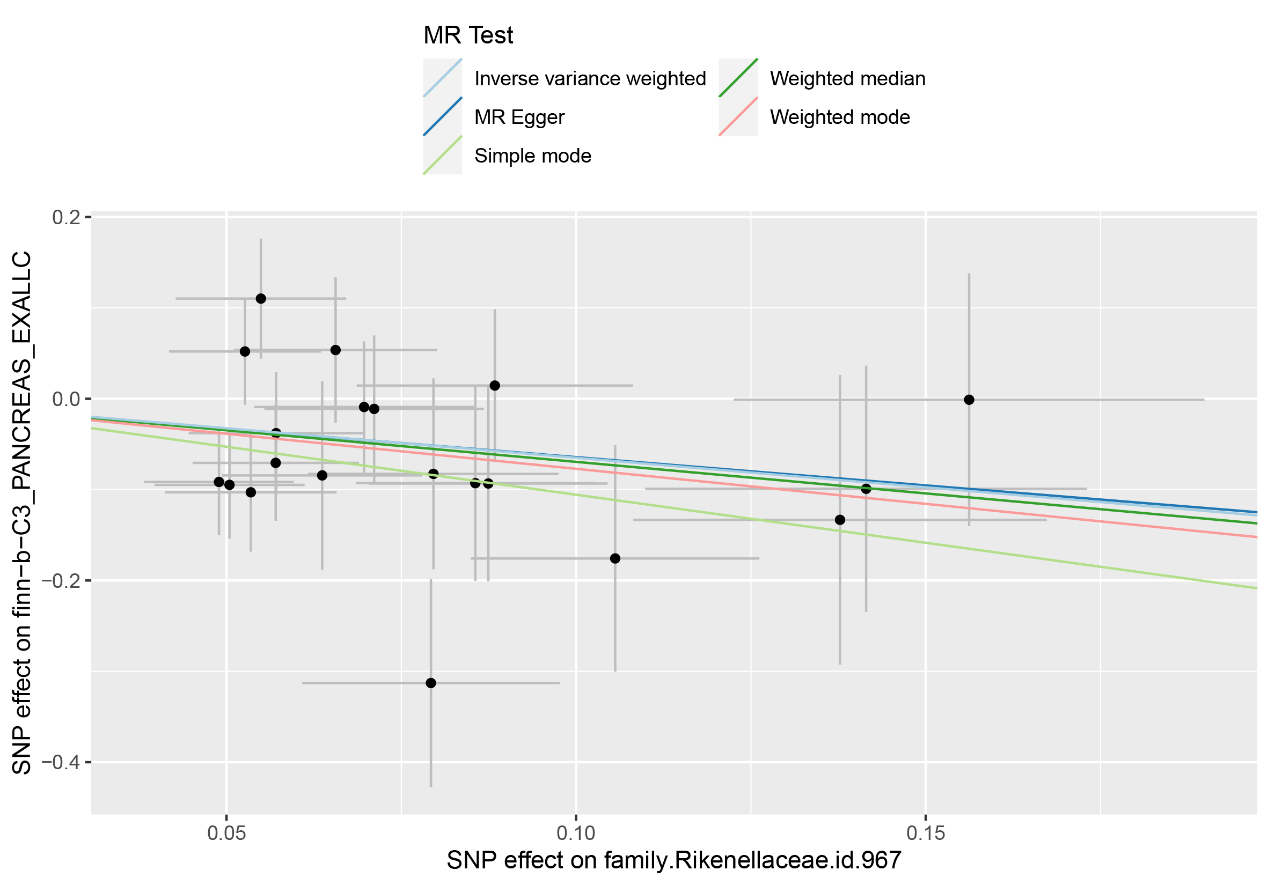


Figure S2.


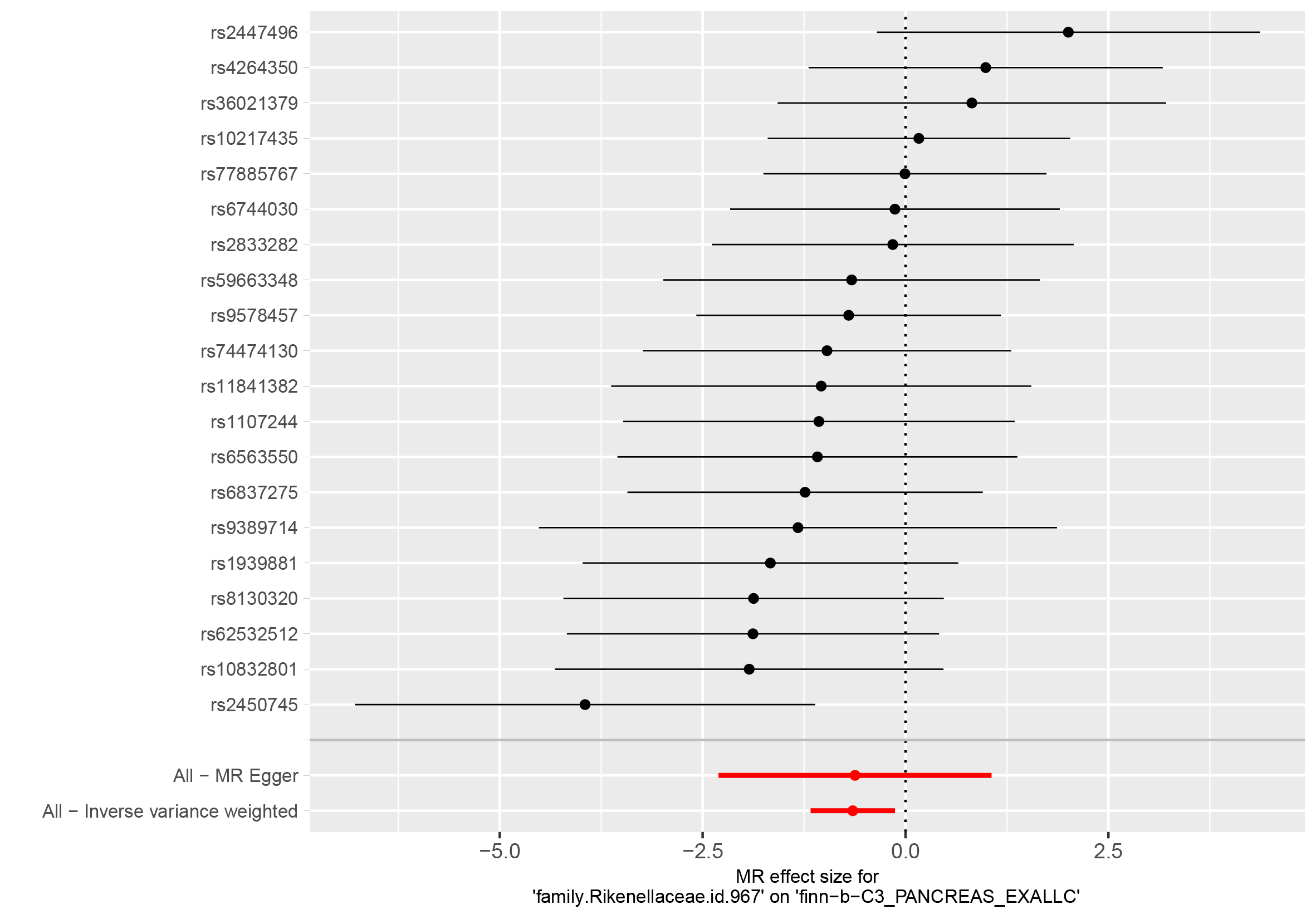


Figure S3.


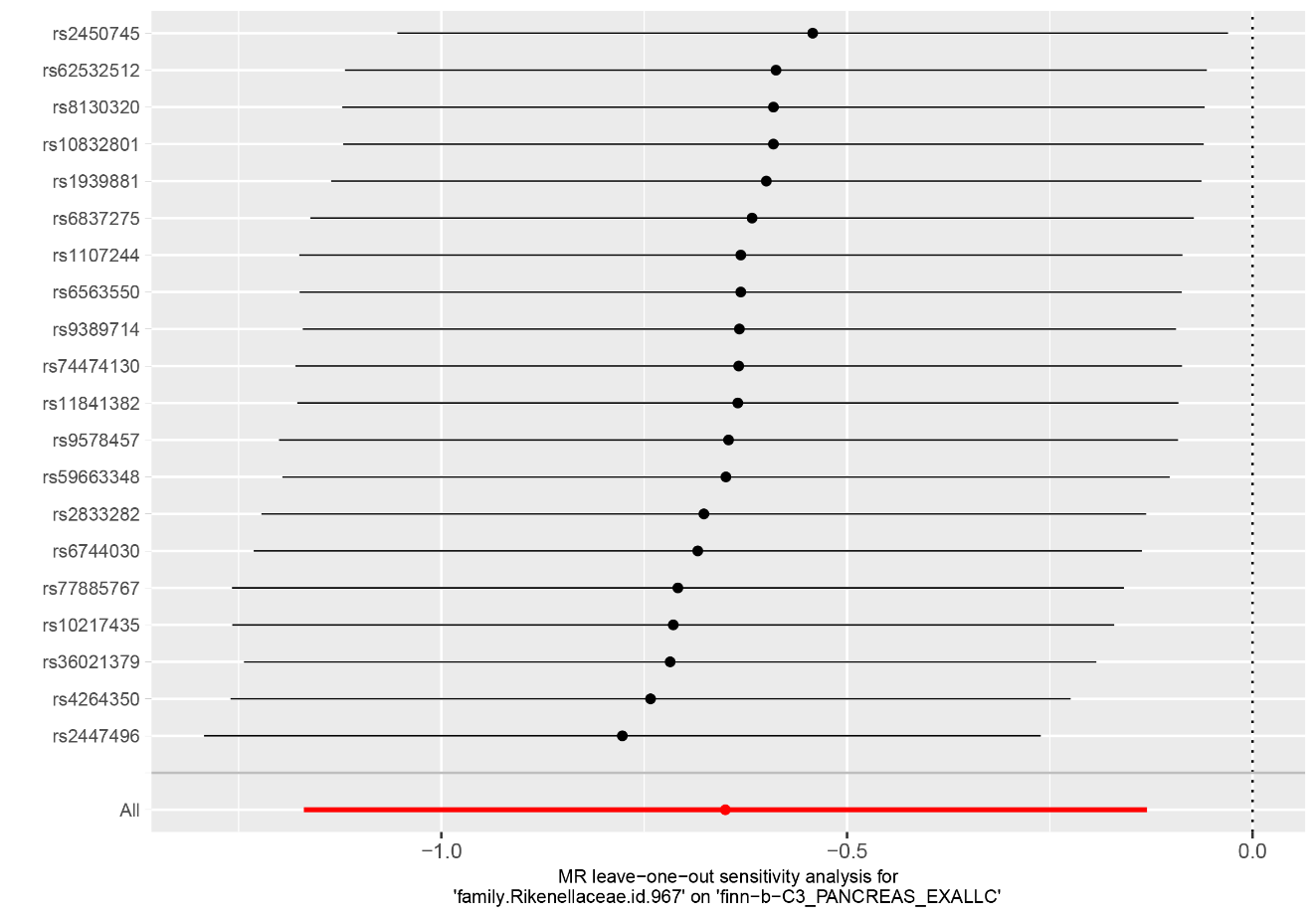


Figure S4.


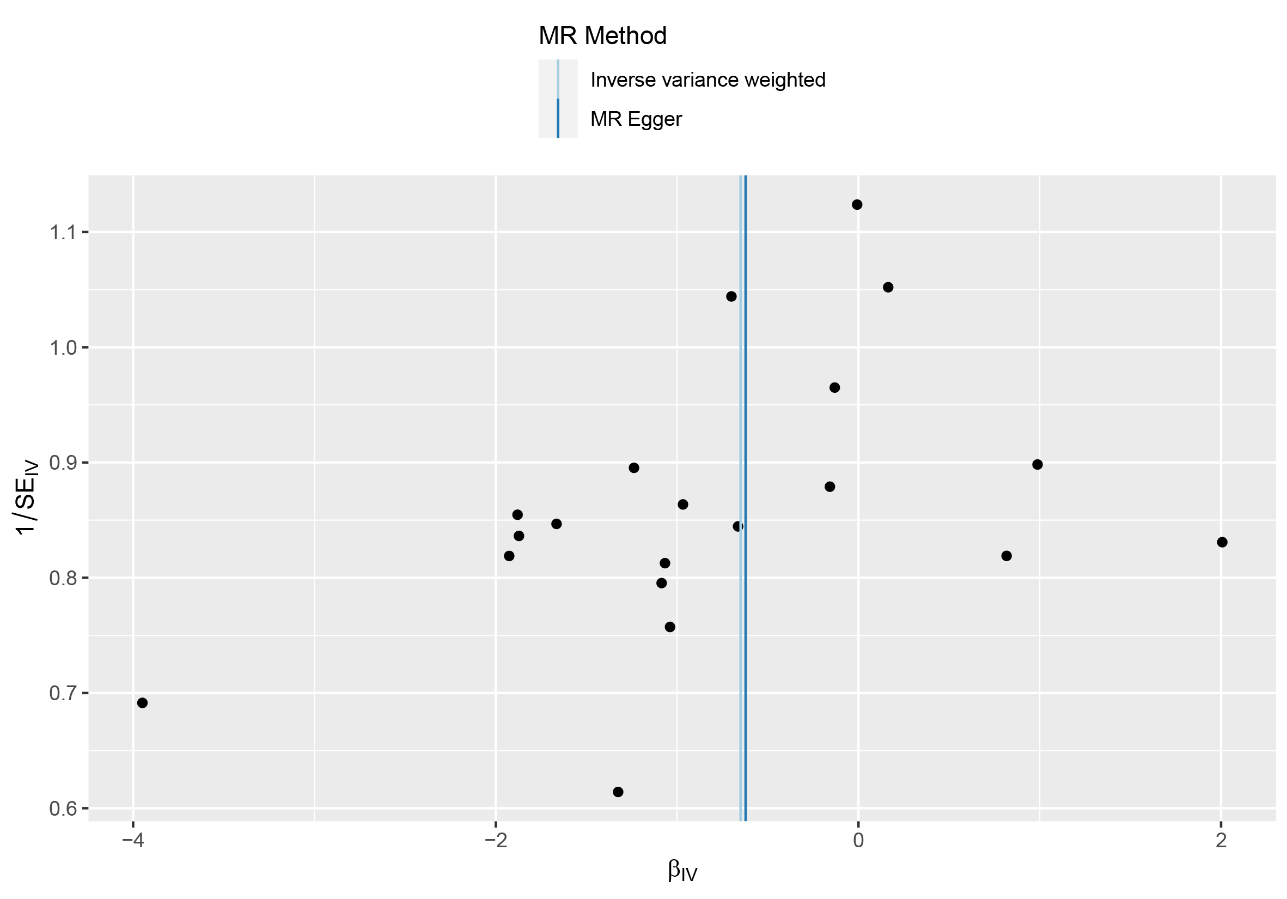

Supplement: Supplementary file 1 — Supporting Information S1 [file SYB2-19-e70027-s001.docx]
